# Supplementary material for: Time-series transcriptome analysis identified differentially expressed genes in broiler chicken infected with mixed Eimeria species
Source: Front Genet. 2022 Aug 8;13:886781. doi: 10.3389/fgene.2022.886781 (PMC9393255; doi:10.3389/fgene.2022.886781)
Supplement: Supplementary file 2 [file DataSheet1.ZIP › 4dpi_KEGG.Gsea.1625100298584/KEGG_VALINE_LEUCINE_AND_ISOLEUCINE_DEGRADATION.html]

Details for gene set KEGG\_VALINE\_LEUCINE\_AND\_ISOLEUCINE\_DEGRADATION[GSEA]

|  || Dataset | TMM\_4dpi\_gct\_format\_4dpi\_gct\_format.Class\_4dpi.cls #PC\_versus\_NC.Class\_4dpi.cls #PC\_versus\_NC\_repos |
| Phenotype | Class\_4dpi.cls#PC\_versus\_NC\_repos |
| Upregulated in class | 0 |
| GeneSet | KEGG\_VALINE\_LEUCINE\_AND\_ISOLEUCINE\_DEGRADATION |
| Enrichment Score (ES) | -0.45073277 |
| Normalized Enrichment Score (NES) | -1.5477086 |
| Nominal p-value | 0.020871986 |
| FDR q-value | 0.16895582 |
| FWER p-Value | 0.8526 |
Table: GSEA Results Summary

  

Fig 1: Enrichment plot: KEGG\_VALINE\_LEUCINE\_AND\_ISOLEUCINE\_DEGRADATION      
 Profile of the Running ES Score & Positions of GeneSet Members on the Rank Ordered List

  

| SYMBOL | TITLE | RANK IN GENE LIST | RANK METRIC SCORE | RUNNING ES | CORE ENRICHMENT || 1 | HMGCS1 | na | 30 | 2.183 | 0.1412 | No |
| 2 | ACAT2 | na | 2179 | 0.392 | -0.0125 | No |
| 3 | ACADS | na | 3016 | 0.280 | -0.0638 | No |
| 4 | HIBADH | na | 3785 | 0.189 | -0.1155 | No |
| 5 | ALDH3A2 | na | 5110 | 0.069 | -0.2215 | No |
| 6 | ACAA2 | na | 5307 | 0.053 | -0.2344 | No |
| 7 | HADH | na | 5702 | 0.017 | -0.2662 | No |
| 8 | ALDH6A1 | na | 7045 | -0.093 | -0.3721 | No |
| 9 | ACAT1 | na | 7276 | -0.116 | -0.3837 | No |
| 10 | HADHA | na | 7423 | -0.129 | -0.3874 | No |
| 11 | AUH | na | 7555 | -0.140 | -0.3891 | No |
| 12 | OXCT1 | na | 7966 | -0.176 | -0.4118 | No |
| 13 | MCCC1 | na | 8068 | -0.186 | -0.4080 | No |
| 14 | MCCC2 | na | 8127 | -0.191 | -0.4003 | No |
| 15 | PCCA | na | 8253 | -0.204 | -0.3973 | No |
| 16 | ACAA1 | na | 8300 | -0.208 | -0.3874 | No |
| 17 | IL4I1 | na | 8390 | -0.217 | -0.3806 | No |
| 18 | DLD | na | 8787 | -0.260 | -0.3965 | No |
| 19 | DBT | na | 8886 | -0.272 | -0.3868 | No |
| 20 | BCKDHB | na | 9427 | -0.339 | -0.4096 | No |
| 21 | HIBCH | na | 9742 | -0.378 | -0.4109 | No |
| 22 | ALDH7A1 | na | 10220 | -0.448 | -0.4213 | Yes |
| 23 | ALDH9A1 | na | 10288 | -0.458 | -0.3967 | Yes |
| 24 | BCAT1 | na | 10469 | -0.491 | -0.3794 | Yes |
| 25 | ACAD8 | na | 10494 | -0.497 | -0.3487 | Yes |
| 26 | HSD17B10 | na | 10557 | -0.509 | -0.3204 | Yes |
| 27 | HMGCL | na | 10631 | -0.525 | -0.2919 | Yes |
| 28 | BCKDHA | na | 10660 | -0.531 | -0.2593 | Yes |
| 29 | EHHADH | na | 10699 | -0.538 | -0.2271 | Yes |
| 30 | IVD | na | 10747 | -0.548 | -0.1950 | Yes |
| 31 | ACADSB | na | 10887 | -0.579 | -0.1685 | Yes |
| 32 | PCCB | na | 11159 | -0.652 | -0.1482 | Yes |
| 33 | ABAT | na | 11359 | -0.728 | -0.1169 | Yes |
| 34 | ECHS1 | na | 11432 | -0.767 | -0.0725 | Yes |
| 35 | MCEE | na | 11546 | -0.834 | -0.0270 | Yes |
| 36 | AOX1 | na | 11728 | -0.999 | 0.0235 | Yes |
Table: GSEA details [plain text format]

  

Fig 2: KEGG\_VALINE\_LEUCINE\_AND\_ISOLEUCINE\_DEGRADATION      
 Blue-Pink O' Gram in the Space of the Analyzed GeneSet

  

Fig 3: KEGG\_VALINE\_LEUCINE\_AND\_ISOLEUCINE\_DEGRADATION: Random ES distribution      
 Gene set null distribution of ES for **KEGG\_VALINE\_LEUCINE\_AND\_ISOLEUCINE\_DEGRADATION**
